# Larval habitat diversity, physicochemical characterization and their effect on the density of larval malaria vectors in the city of Accra, Ghana

**DOI:** 10.21203/rs.3.rs-4953028/v1

**Published:** 2024-09-20

**Authors:** Abdul Rahim Mohammed Sabtiu, Isaac Amankona Hinne, Isaac Kwame Sraku, Richard Tettey Doe, Simon Kwaku Attah, Fred Aboagye-Antwi, Yaw Asare Afrane

**Affiliations:** University of Ghana; University of Nevada; University of Ghana; University of Ghana; University of Ghana; University of Ghana; University of Ghana

**Keywords:** Anopheles, larvae, urban, habitats, An. stephensi, Ghana, Irrigation, physicochemical, polluted

## Abstract

**Background:**

Malaria is more prevalent in rural areas due to fewer mosquito breeding habitats in urban settings. However, urban factors such as irrigated farming, open sewers, and discarded containers create mosquito breeding sites. This study investigates the diversity and distribution of larval habitats and the impact of physicochemical characteristics on the presence and density of *Anopheles gambiae* s.l. larvae in Accra, Ghana.

**Methods:**

Larval surveys and collections were conducted at fifteen locations in Accra, divided into five categories: Irrigated Urban Farming (IUF), Lower Socioeconomic Status (LS), Middle Socioeconomic Status (MS), High Socioeconomic Status (HS), and Peri-urban (PU) areas. Physicochemical parameters were measured, and species identification was performed using morphological and molecular methods.

**Results:**

A total of 727 breeding habitats were identified, with 65.34% (475/727) positive for Anopheles larvae. Drainage ditches were the most common habitat type (48.21%, 229/475). The highest abundance of *An. gambiae* s.l. was found in IUF sites (6,244/22,919), especially during the rainy season (77.01%, 17,650/22,919; R^2^ = 3.46, P = 0.000). Polluted habitats, including household effluents, had higher ammonium levels (3.4 mg/L NH -N) compared to unpolluted ones (1.3 mg/L NH -N). Other distinguishing parameters included dissolved oxygen (34% vs 52.9%), conductivity (5106 μS/cm vs 2049 μS/cm), and total dissolved solids (3181 mg/L vs 1255 mg/L). The predominant malaria vector was *An. coluzzii*(54.4%, 368/677). Additionally, the invasive *An. stephensi,* previously unreported in Ghana, was detected.

**Conclusion:**

Malaria vectors breed in diverse and often polluted urban habitats, with high larval densities in urban agricultural areas. The detection of the invasive *An. Stephensi* highlights the need for continuous monitoring and vector control strategies in urban settings.

## Background

Malaria, traditionally considered a rural disease, is less prevalent in urban areas primarily due to the scarcity of preferred breeding habitats for malaria vectors. However, certain urban factors, such as irrigated farming, create year-round breeding sites for mosquitoes (1)(2). Urban agriculture, a crucial socioeconomic activity that help to provide income and food security for urban residents(3)(4), but inadvertently supports mosquito breeding, thus posing a significant public health challenge.

Moreover, the existence of broken and open sewers, as well as discarded containers such as tin, can, plastic bowls, buckets, car tyres and other water impoundments, potentially serve as breeding habitats for mosquitoes in urban settings. Construction of roads, residential and other structures as a result of unplanned urbanization has also been implicated to contribute to the creation of breeding habitats (5).

In Africa, urbanization is increasing at an alarming rate (6). The African urban population is envisaged to escalate by 50% and 60% by the year 2030 and 2050 respectively (7). In Ghana, the high rate of infrastructural development is a reflection of what is observed in Africa, with the city of Accra recording the highest urbanization rate among other cities in the country (8). This rapid unplanned urbanization could have major implications on the transmission of malaria within the city as the dynamics of urbanization have been shown to be strongly associated with malaria vector abundance (9).

However, there is paucity of data on urban malaria transmission in the city of Accra, especially on the vectors involved (1)(10). The few studies conducted hitherto did not provide data on larval habitat types, and distribution and abundance of larval malaria vectors in the different urban areas of Accra(11)(12). This study, therefore, aims to investigate the larval habitat diversity and distribution, and the effect of their physicochemical characteristics on the presence and density of *Anopheles gambiae* s.l. larvae in Accra, Ghana.

## Methodology

### Study sites

**Figure 1:**
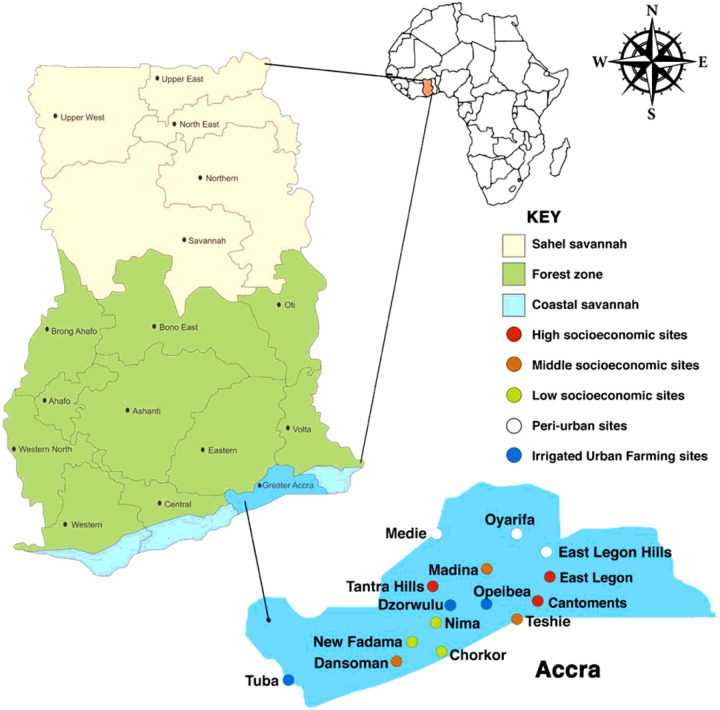
Map of Ghana and Accra showing location of the study sites.

The study was undertaken in fifteen (15) sites within the city of Accra, the capital of Ghana, and peri-urban areas. Three (3) study sites were randomly selected in Accra, from areas that were categorized as low socio-economic (LS), Middle socioeconomic (MS), High socioeconomic (HS) and, Irrigated urban farming (IUF) areas. In comparison three (3) independent sites were also selected from the peri-urban areas surrounding Accra (Fig. 1). It is hypothesized that there are more mosquitoes in peri-urban and rural areas than urban areas.

In the irrigated urban farming categories, the sites included were Opeibea (5°35’52.8”N 0°10’48.2”W), Dzworwulu (5°36’53”N 0°12’03”W), and Tuba (5° 30’47”N 0° 23’ 16”W). Larval habitats are created all year round in these sites due to regular irrigation. Moreso, different agricultural chemicals that could contribute to insecticide resistance in mosquitoes are used by the farmers in these farms. Sites for the lower Socioeconomic (LS) consisted of Nima (5° 35’ 0” N, 0° 12’ 0” W), Chorkor (5°31’39”N 0°13’55”W), and New Fadama (5°35’54.1”N 0°14’52.2”W). These are slums, where the inhabitants reside in outmoded and overcrowded structures located in poorly demarcated plots of land. These areas have no well-designed drainage systems, with very poor sanitation systems. Madina (5°41’0”N 0°10’0”W), Dansoman (5° 33’ 0” N, 0° 16’ 0” W), and Teshie (5° 35’ 0” N, 0° 6’ 0” W) were selected as the Middle Socioeconomic (MS) sites. This category represents areas that have standard housing structures built on well-demarcated plots of land, with majority having access to either tarred or untarred road network. Areas within this category also have well-designed drainage and sanitation systems but poorly managed. East Legon (5°38’16.39”N 0°9’40.33”W), Cantonment (5° 35’ 10” N, 0° 10’ 35” W) and Tantra Hill (5° 34’ 44.0148”N 0°13’ 46.812”W) categorized as High Socioeconomic (HS) status sites have housing structures that are of the highest quality in Ghana. These sites have a well-planned environment with best-managed drainage and sanitation systems in the country. They have very good road network with huge sections of the roads tarred, hence have few pot-holes to serve as breeding habitats. The peri-urban areas consisted of Oyarifa (5° 46’ 14” N, 0° 10’ 50” W), Medie (5° 45’ 43”N 0° 19’ 20”W), and East Legon Hills (5° 41’ 28.5468”N 0° 6’ 0.018”W). These are areas just outside the city with many new settlements. Most of the roads and buildings are under construction with higher vegetation cover. During the rainy season numerous breeding habitats are created due to the nature of the land in these areas. The differences in landscape, drainage systems, sanitation, and land use (agriculture/non-agriculture) will help to compare and understand their impact on urban malaria transmission and vector resistance status to insecticides.

Accra which lies within the Greater Accra region, had a total population of about 2,557,000 in 2021, a population growth rate of 24.13% from 2010 to 2021 and a land area of 225.67 km^2^ (Ghana Population and Housing Census, 2021). The region has two rainy seasons with average annual rainfall and temperature of 730 mm and 27.6 °C respectively. The relative humidity is mostly high, ranging from 65% during the midafternoon to 95% at night. The rainfall pattern coupled with the poor drainage system supports the formation of stagnant waters, whereas the temperature and humidity create a favourable environment for mosquitoes.

### Larval habitat characterization, abundance and measurement of densities

All larval habitats encountered were classified into natural or artificial. Natural habitats included natural ponds, swamps, and streams, while drainage ditches, hoof prints, footprints, car tyres, wells and furrows were categorized as man-made habitats. Land-use type was also classified based on the activities taking place on the land where larval habitat is located and natural vegetation. These included compounds with human settlements, farmland for cultivation sites, and roads and swamps. The habitat length and width were measured and recorded in metres. The vegetation cover was visually estimated as the percentage of vegetation covering the water surface. This was recorded as zero (0) where vegetation was not found on the surface of the habitat, ≤ 24%, 25–49%, 50–74%, and 75–100% surface coverage(13)(14).

Larvae were collected from all fifteen study sites in 2022 during the dry (from February to March) and rainy (from June to July) seasons. The WHO standard dipping method using the 350ml standard dipper was used to sample larvae from all potential larval habitats. Habitat sizes were categorized into ≤ 1, >1, > 2–5, >5–10, > 10–100, or > 100m and a maximum dip of 2, 4, 6, 10, 50, and 150 were taken respectively (depending on habitat size). For habitats with much smaller sizes such as footprints and hoofprints, a ladle was used to collect the samples. Anopheles larval instar stages collected were classified as (L1-L2) and (L3-L4), and the total number was estimated. Larvae and pupae collected were recorded, and larval density was calculated as the ratio of the number of larvae collected per number of dips taken(15)(2). The geographical coordinates of each larval habitat were recorded using a GPS device (Garmin eTrex 10 Worldwide Handheld GPS Navigator). Sampling usually started early in the morning (between 6:00 and 10:00 hours GMT) and early in the evening (between 15:30 and 18:00 hours GMT) to prevent distraction from the reflection of the sun on the water’s surface. Whenever there was heavy rain, larval sampling was halted and continued after three days.

### Measurement of physical and chemical parameters of larval habitats

The physicochemical characteristics of the larval habitat were also measured using the PC60 Premium Multi-Parameter Tester (Apera Instruments, LLC). The recorded parameters included pH, temperature, salinity, specific conductivity (SPC), and total dissolved solids (TDS).

Additionally, some larval habitats, identified as either polluted or unpolluted (relatively clean visually) during larval collection, were selected for further physicochemical measurement. These habitats were located in Nima, Chorkor, Teshie and Madina. The YSI Pro Quatro Multiparameter Meter (YSI Incorporated, USA) was employed to measure temperature, pH, dissolved oxygen (DO), specific conductivity (SPC), salinity, total dissolved solids (TDS), volume/bulk resistivity (RES), ammonia (NH3-N), and ammonium (NH4-N) levels in these habitats.

### Mosquito species identification

Samples (larvae and pupae) collected from the field were put into plastic containers and transported to the insectary of the Department of Medical Microbiology, University of Ghana, and raised into adults. Pupae were picked into pupal cups using Pasteur pipette and placed into mosquito cages daily. Larvae were fed with Tetramin^®^ fish meal and emerged adults immediately fed on a 10% sugar solution soaked in a clean cotton wool ball. Adult mosquitoes were kept at a temperature and relative humidity of 27 ± 2°C and 72 ± 5% respectively. Emerged adults were aspirated into paper cups covered with muslin nets, and killed/knockdown using chloroformed. Mosquitoes were then identified morphologically under a stereomicroscope (Olympus, SZ60, Japan) using the keys developed by Maureen Coetzee(16). Sibling species of *Anopheles gambiae* s.l. were identified using rDNA polymerase chain reaction (PCR) (17). *An. gambiae* s.s. and *An. coluzzii* were further identified using PCR-restriction fragment length polymorphism (RFLP)(18).

### Data analysis

Descriptive analyses were done to compare the abundance of the various larval breeding habitat types and larval densities in the different study sites and seasons. This was presented in the form of tables and graphs. The larval densities were calculated as the total number of larvae per dip. The total number of dips for smaller habitats such as foot and hoofprints were assumed as one dip. The density of Anopheles mosquito larvae was compared among the various breeding habitats and study site categories. The Mann-Whitney U test and the Kruskal-Wallis test were used to test the associations between continuous and categorical variables. The chi-square and Fisher’s exact tests were used to test the association between two categorical variables. Logistic regression using the glmer (from the lme4 package) was used to test the association between the habitat characteristics with categorical data and the presence of Anopheles larvae. The forward-backward stepwise method was used to select the best model based on Alkalie Information Criterion (AIC). Nested generalized linear mixed models using AD model builder with study sites nested within site class using the glmmADMB package was used to model the effect of habitat characteristics on larval densities. Correlations between physicochemical variables and larval densities were assessed using Pearson’s correlation analysis and principal component analysis (PCA). All statistical analyses were done in R 4.2.2 via RStudio (2022.12.0 + 353).

## Results

### Larval habitat type, distribution and abundance

A total of 727 breeding habitats were found in all the fifteen study sites, of which [63.41%, n = 461/727] were positive for *Anopheles* larvae (Table 2). Out of the total habitats that were positive for *Anopheles* larvae, [27.33%, n = 126/461] were found in the dry season and [72.67%, n = 335/461] in the rainy season (Table 2). Breeding habitats such as car tires, hooves prints and puddles were only found during the rainy season (Table 1). Overall, the most abundant larval habitat types that were encountered during the survey include drainage ditches [49.38%, n = 359/727], followed by tire tracks [14.17%, n = 103/727], swamps [11.55%, n = 84/727], furrows [6.74%, n = 49/727], artificial pond [5.64%, n = 41/727], foot imprint [4.26%, n = 31/727], puddles [2.89%, n = 21/727], well [2.48%, n = 18/727], natural ponds [1.38%, n = 10/727], car tires [0.69%, n = 5/727], hoofprints [0.55%, n = 4/727], and then pits [0.03%, n = 2/727], (Table 1).

A higher proportion of larval breeding sites were identified in the irrigated urban farming (IUF) site category (24.76%, 180/727), followed by middle socioeconomic (MS) 158 (21.73%), peri-urban (PU) 155 (21.32%), high socioeconomic (HS) 153 (21.05%), whereas the lowest socioeconomic (LS) category recorded the lowest (9.22%, 67/727) proportion of breeding sites. Furrows, natural and artificial ponds were only found in IUF sites. Drainage ditches were the most abundant habitat type encountered and was significantly associated with site category (χ^2^ = 228.13, df = 32, *P*< 0.001).

### Seasonal distribution and densities of larval malaria vectors

A total of 30,552 mosquitoes belonging to two different genera were collected from all the study sites during the sampling period; HS [n = 5,533/30,552, 18.1%], PU [n = 4,811/30,552, 15.7%], MS [n = 5,970/30,552, 19.5%], LS [n = 5,843/30,552, 19.1%], IUF [n = 8,395/30,552, 27.5%]. Overall, 75.02%, (22,919/30,552) of mosquitoes sampled were *Anopheles,* whereas 24.98%, (7,633/30,552) were *Culex*.

Throughout the study period, the abundance of *Anopheles* was highest in IUF [6244/22,919], followed by MS [ 4,557/22,919], LS [4,505/22,919], HS [4,097/22,919], then PU [3516/22,919]. Overall, *An. gambiae* s.l. were more abundant in the rainy season (77.01%; 17,650/22,919) than in the dry season (22.99%; 5,269/22,919). The mean abundance of *An. gambiae* was significantly associated with season (*R*^2^ = 3.46, *P=* 0.000).

The site category with the most abundant culicine larvae sampled was from IUF = [28.2%, 2,151/7,633], followed by HS = [18.8%, 1,436/7,633], MS = [18.5%, 1,413/7,633], LS [17.5%, 1,338/7,633] and then PU = [16.9%, 1295/7,633]. Regression analysis indicated a significant productivity effect of *Culex* larvae in breeding habitats on the presence of *Anopheles* larvae (*R*^2^ = 2.78, *P=* 0.001). Similarly, generalized linear models analysis indicated that the presence of *Culex* larvae in breeding habitats had a significant effect (B = −0.46340, p= 0.001) on *Anopheles* larval density.

A high abundance of *Anopheles* larvae 8,560 (37.35%) were collected from drainage ditches whereas car tires recorded the lowest 78 (0.34%). There was a significant association between habitat type and the presence of *Anopheles* larvae (χ^2^ = 22.721, df = 8, P= 0.004).

The highest larval densities of 19.22 and 13.22 larvae/dip were recorded in swamps (MS) and tire track (IUF) respectively in the rainy season. However, in the dry season the highest larval density, 12 larvae/dip was recorded in tire track (IUF) (Table 3). Larval habitats with sizes less than 10 meters had significantly higher larval densities compared to those with sizes between 10 to 100 meters (*X*^2^ = 6.41, *df*= 1, *P*= 0.01). *Anopheles gambiae* s.l. larval density was significantly associated with season (*t*= 4.14, *P*= 0.00). The student T-test analysis indicated a significant association between *Anopheles gambiae sl* larval density and site category (*t*= 2.58, *P*= 0.01). Similarly, larval density was significantly associated with the presence of algae (*Z*= −2.19, *P*= 0.03), land use type (t =−1.93, *P*=0.053).

### Physical and chemical properties of larval habitats

Overall, the pH of larval habitats in the study sites ranged from 7.0 to 9.02. The lowest pH was recorded from a drainage ditch (MS category) and the highest in a swamp (MS category) during the rainy season (Table 5b). A high (4940 ppm) (LS) turbidity was recorded in a swamp, whereas, the lowest (1.08 ppm) was recorded in a tyre track (IUF). The highest salinity (2.2ppt; dry season) (Table 4a) and conductivity (4033 mS/cm; rainy season) (Table 4b) were measured in natural pond (PU) and well (LS) respectively. The lowest salinity (0.07ppt) and conductivity (124.1 mS/cm) were both recorded in a natural pond respectively during the dry season in an IUF site category (Table 4a).

**Figure 2:**
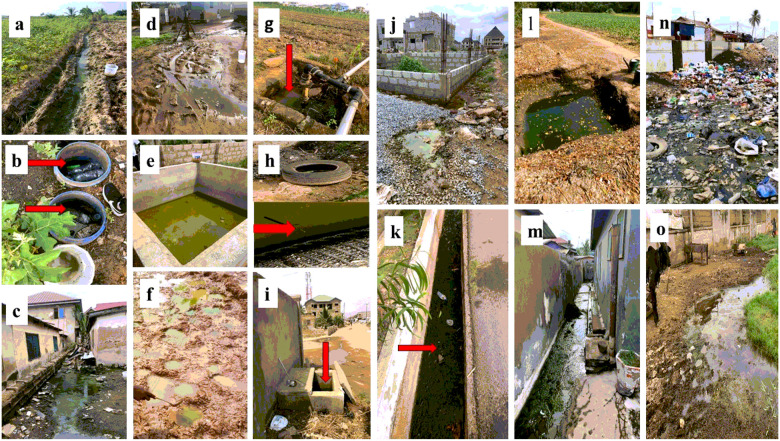
Types of habitats found during the study period. a furrows, b plastic containers, c puddle on cemented floor, d puddle in a compound, e concrete tank, f hoofprints, g base of water pumping machine, h car tyre, i soakaway cement tank, j puddle at construction site, k concrete drainage ditch, l artificial pond for irrigation, m puddle on cemented floor n drainage ditch and dumping site o puddle in cattle range.

The relationship between the physicochemical variables and *Anopheles* larval productivity was determined using the principal component analysis. The projections showed that larval productivity was linked to the variation of some physicochemical parameters (Fig. 2). Correlation analysis (PCA) between the physicochemical parameters and distribution of *Anopheles* mosquitoes is represented in Table 5. The result shows a non-significant negative correlation (r= −0.058) between pH and *Anopheles* larval densities. Similarly, the abundance of *An. gambiae* s. l. showed no correlation (*r=* 0.090) to habitat temperature. However, there was a strong positive correlation between some of the chemical parameters measured in the larval habitats; EC and salinity (r = 0.907), EC and TDS (r = 0.883), and Salinity and TDS (r = 0.908) (Table 5).

### Physical and chemical parameter levels in polluted and unpolluted habitats

The physical and chemical properties of polluted and unpolluted habitats were measured over a period of four (4) weeks. Polluted habitats had lower dissolved oxygen (1.4–3.6 ppm) and higher Total Dissolved Solids (1,026.5–4,567.0 mg/L) compared to unpolluted habitats (DO: 2.0–7.8 ppm; TDS: 751.0–1,780.2 mg/L). In polluted habitats, salinity (0.8–3.9 ppt) and specific conductance (1.6–8.0 mS/cm) were higher than in unpolluted habitats (salinity: 0.6–1.4ppt, specific conductance: 1.2–2.7 mS/cm). Polluted habitats also had elevated ammonium (0.1–11.9 mg/L) and ammonia (0–1.6 mg/L) levels and higher pH (8.3–9.1) values compared to the unpolluted habitats (ammonium: 0–4.9 mg/L), ammonia: 0–0.2 mg/L) pH (7.8–8.3) (Table 6).

Simple linear regressing analysis indicated that larval abundance had a significant relationship with polluted larval habitats: Nima (2.6 vs. 1.2), Chorkor (6.6 vs. 4.5), Teshie (4.7 vs. 1.9), and Madina (3.1 vs. 1.7). (B = 4.25, *P=* 0.002). Correlation analysis revealed a strong positive association between larval abundance and some physicochemical parameters: specific conductance (SPC) (R = 0.261), conductivity (COND) (R = 0.253), salinity (SAL) (R = 0.240), total dissolved solids (TDS) (R = 0.252), resistivity (RES) (R = 0.610), and pH (R = 0.710). Although ammonium (R = −0.4131, P = 0.309) and ammonia (R = −0.159, P = 0.706) showed non-significant negative correlations, a strong positive and significant correlation was found between larval abundance and pH (R = 0.710, P = 0.048).

### Species discrimination in the Anopheles gambiae complex

A subsample of 679 of *An. gambiae* s.l. from all the study sites were randomly selected and used to discriminate the sibling species. *An. Coluzzii* 54.79% (372/677) was the most abundant species followed by *An. gambiae* s.s 42.97% (285/679) and *An. gambiae/An. coluzzii* Hybrid 2.81% (19/679). About 0.44% (3/679) were detected to be *An. stephensi*. According to the site category, *An. coluzzii* was the abundant species sampled in all the various categories, except in the HS = 111[An. *gambiae s.s*. = 59, *An. Coluzzii* = 50, hybrid = 2] and PU = 150 [*An. gambiae s.s*. = 111, *An. coluzzii=* 30, hybrid = 9] sites, where the species were dominated by the *An. gambiae* s.s. The new invasive species, *An. stephensi* was found in Dansoman, Tuba and Nima which are within the MS, IUF and LS category respectively (Table 7).

## Discussion

Investigating malaria vector breeding habitat types, habitat characteristics and *Anopheles* larval abundance in urban settings is an essential element of urban malaria control (9). This can help in the elimination of urban malaria through effective larval source management. The present study assessed the larval habitat types, characteristics and their effect on the density of *Anopheles gambiae* s.l. in Accra, Ghana. Twelve (12) different larval habitat types, including drainage ditches, car tyres, and puddles were found. Irrigated urban farming (IUF) and Peri-urban (PU) sites had the highest frequency of larval habitats in dry and rainy season respectively. Drainage ditches were the most abundant and most productive habitat type for *Anopheles* mosquito larvae. *Anopheles coluzzii* is the most dominating malaria vector species found. This study identified the invasive malaria vector, *An. Stephensi* for the first time in Ghana.

The most abundant habitat type was drainage ditches which carried effluents from people’s homes. Other habitats such as car tires, hoof-prints and puddles were usually formed during the rainy season. Most of the mosquito breeding sites encountered during the survey in the different site categories resulted from anthropogenic activities, and their characteristics were related to factors associated with urbanization and agriculture. This emphasizes the importance of human activities through land-use in the creation of *Anopheles* breeding habitats and the impact they have on malaria transmission. Similar observation was reported in Accra and Takoradi (12), and in Cape Coast (19), Ghana, highlighting that man-made breeding habitats were the most abundant. The presence of numerous drainage ditches functioning as breeding habitats may stem from inadequate sanitation practices and insufficiently regulated urban development by both public authorities and residents. This results in the obstruction of drainage systems, allowing water to accumulate for extended periods and creating suitable conditions for mosquito breeding. In contrast, other studies highlighted puddles as the predominant breeding grounds for *Anopheles* mosquitoes in urban settings (12)(20).

Even though there was seasonal variation in larval habitat abundance with higher abundance recorded in the rainy season, breeding habitats were found throughout the dry and rainy seasons across all site categories. This could potentially contribute to malaria transmission all year round (21)(2). The IUF site category had the highest number of breeding habitats, likely attributable to the predominantly lowland nature of most IUF sites and their regular irrigation practices. This finding corroborates with that of Afrane *et al*. (22) and Klinkenberg *et al*. (11). Their study reported that irrigated fields generated large numbers of mosquitoes. These findings were similar to other studies in the town of Niono, Mali by Diuk-Wasser *et al*. (24) and in Dar es Salam, Tanzania by Dongus *et al*. (25) Ghana by Hinne *et al*. (2), they reported that irrigated farms contribute to high abundance of malaria mosquitoes. Furthermore, the IUF site category had the most diverse breeding habitat types, which included eight of the eleven habitat types encountered, coinciding with high abundance of *An gambiae* larvae reported in this study.

Throughout the entire sampling period, *An. coluzzii* emerged as the most predominant species, potentially influenced by the permanent nature of most of the breeding habitats. This finding aligns with previous reports by Kudom *et al*. (19) and Hinne *et al*. (2), indicating its preference to breeding in permanent larval habitats such as irrigated fields (26). Its abundance and distribution remain consistent irrespective of seasons or rainfall patterns, allowing for year-round breeding in various habitats. Similar findings were reported by Chabi *et al*. (27) in Ghana and Ossé *et al*. (28) in Benin.

Larval densities were observed to be higher during the rainy season in all sites, however, higher mean larval densities were observed in irrigated urban farm (IUF) site categories during both seasons. Additionally, the current study indicated that higher larval densities were significantly associated with habitat size, season of sampling, presence of algae in breeding habitats, land use type, and site category. Various physical, chemical, and biological parameters have the potential to influence habitat productivity and larval distribution in different breeding habitats. Previous studies, by Onchuru *et al*. (29), Hessou-Djossou *et al* (20) and Forson *et al*. (14), have reported a positive relationship between *Anopheles* larval density, and some physicochemical properties such as vegetation cover, habitat size, temperature, pH and conductivity. Muturi *et al*. (30) suggested that low water temperatures result in a decline in the growth of microorganisms that serve as food for mosquito larvae.

Findings from the current study showed that temperature and pH was negatively correlated with habitat preference of *Anopheles* mosquitoes in the study area. This conformed to the findings of Akeju *et al*. (31) and Getachew *et al*. (32); they both reported that pH of the habitat of immature stages of *Anopheles* mosquito was not significantly correlated with the density of *Anopheles* larvae in their respective location of study, though some other species of mosquito larvae have been reported to show a significant correlation with pH (33)(34).

More importantly, the data suggests that polluted *Anopheles* larval habitats in the study sites have higher levels of dissolved ions, salinity, total dissolved solids, and ammonia, as well as lower dissolved oxygen levels and resistivity compared to unpolluted habitats. These differences in physicochemical parameters coupled with the high abundance of *Anopheles* mosquito larvae could potentially influence the suitability of these habitats, hence affect Larval Source Management (LSM) and the dynamics of malaria transmission in urban settings.

## Conclusion

Malaria vector species were identified breeding in diverse habitat types. Urban agricultural areas had the highest larval densities. Additionally, this study uncovered the presence of the new invasive malaria vector, *An. stephensi*. This discovery holds significant public health implications as this mosquito species demonstrates high resistance to chemical-based vector control and possesses highly invasive characteristics. To address these challenges, Larval Source Management (LSM) should be adapted to encompass all potential mosquito breeding sites in urban areas, especially those with agricultural activities.

## Figures and Tables

**Figure 3:**
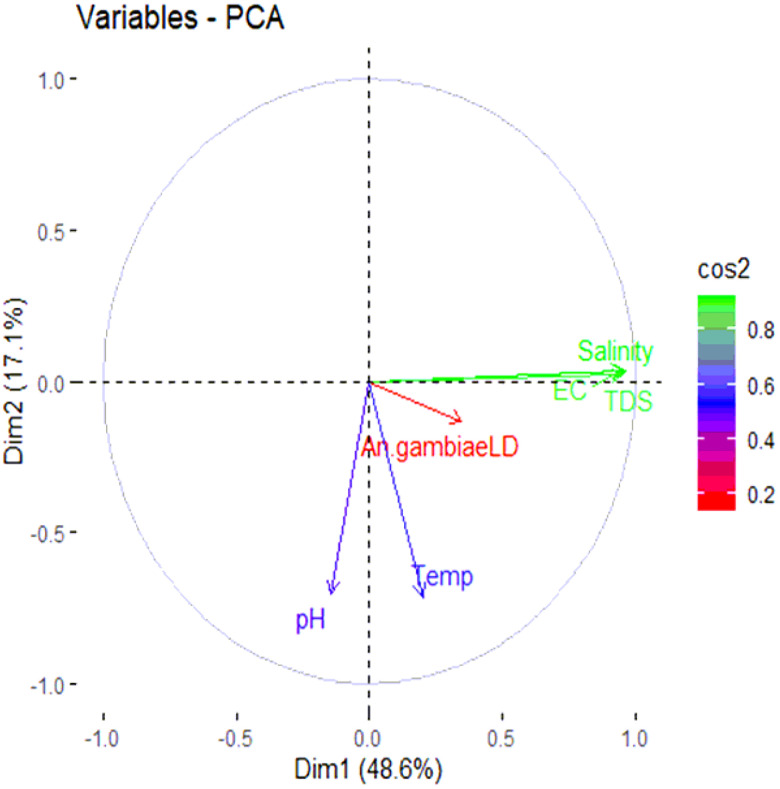
Figure 2: Contribution of physicochemical parameters to larval abundance

**Table 1:** Larval habitat types and the presence of *An. qambiae* s.l during the dry and wet seasons

Habitat type	Breeding habitats N (%)		Habitats with mosquito larvae present N (%)		Habitats with Anopheles species present N (%)	
	Dry	Wet	Dry	Wet	Dry	Wet
Artificial pond	25 (9.51)	16	20	11	17	9
Car tyre	0	5	0	5	0	4
Drainage ditch	168	191	99	139	84	134
Footprint	1	30	1	25	1	24
Furrow	35	14	19	12	8	12
Hoofprint	0	4	0	3	0	3
Natural pond	7	3	7	2	4	2
Pit	2	0	2	0	2	0
Puddle	0	21	0	11	0	11
Swamp	9	75	8	54	5	52
Tyre track	4	99	3	79	3	78
Well	12	6	3	6	2	6
Total	**263**	**464**	**162**	**347**	**126**	**335**

**Table 2:** Mosquito larval abundance and distribution in all site categories

Site Class	*An. gambiae* s.I. N (%)		*Culex*	Total
	Dry Season	Wet Season		
High Socio-economic (HS)	550 (10.44)	3547 (20.10)	1436 (18.8)	5533 (18.11)
Irrigated Urban Agriculture (IUF)	1835 (34.83)	4409 (24.98)	2151 (28.2)	8395 (27.48)
Lower Socio-economic (LS)	922 (17.50)	3583 (20.30)	1338 (17.5)	5843 (19.12)
Middle Socio-economic (MS)	1056 (20.04)	3501 (19.84)	1413 (18.5)	5970 (19.54)
Peri-urban (PU)	906 (17.01)	2610 (14.79)	1295 (16.9)	4811 (15.75)
**Total**	**5269 (100)**	**17650 (100)**	**7633 (100)**	**30552 (100)**

**Table 3:** *Anopheles* larval density in the dry and rainy seasons

Habitat type	Larval density (larvae/dip)									
	HS		IUF		LS		MS		PU	
	Dry	Wet	Dry	Wet	Dry	Wet	Dry	Wet	Dry	Wet
Artificial pond	0	0	4.34	0.78	0	0	0	0	0	0
Car tyre	0	0.42	0	0	0	3.00	0	2.17	0	0
Drainage ditch	1.66	1.11	4.13	0.64	4.69	4.20	1.98	2.70	1.86	5.02
Footprint	0	0	0	2.78	0	2.51	0	4.75	0.27	0.67
Furrow	0	0	1.61	5.18	0	0	0	0	0	0
Hooves print	0	0	0	0	0	0	0	0	0	2.00
Natural pond	0	0	4.59	0.42	0	0	0	0	1.38	0.23
Pit	0	0	6.12	0	0	0	0	0	0	0
Puddle	0	1.04	0	3.89	0	0	0	2.68	0	0.14
Swamp	0	0.87	4.24	0.88	0	2.60	0	5.04	6.17	1.37
Tyre track	0	1.64	12	8.77	0	3.97	0.61	2.72	0	1.56
Well	0	0.25	0.94	0	1.25	4.0	0	1.07	0	1.67

**Table 4 a:** Physicochemical characteristics of larval habitat type and site categories during the dry season

	pH					Temperature (°C)					Salinity (ppt)					TDS/Turbidity (ppm)			
Habitat type	LS	MS	HS	PU	IUF	LS	MS	HS	PU	IUF	LS	MS	HS	PU	IUF	LS	MS	HS	PU
**Artificial pond**	-	-	-	-	7.48	-	-	-	-	28.05	-	-	-	-	0.37	-	-	-	-
**Natural pond**	-	-	-	7.51	7.65	-	-	-	26	26.80	-	-	-	0.05	0.07	-	-	-	86.5
**Pit**	-	-	-	-	7.5	-	-	-	-	29.45	-	-	-	-	0.59	-	-	-	-
**Drainage ditch**	7.2	7.34	7.31	7.33	7.48	27.2	26.5	26	26.1	25.22	1.36	0.32	0.31	0.35	0.30	956	418	419	324
**Swamp**	-	-	-	7.26	7.46	-	-	-	27	29.14	-	-	-	0.2	0.22	-	-	-	168
**Well**	7.08	-	-	-	7.34	29	-	-	-	29.50	2	-	-	-	0.18	285	-	-	-
**Footprint**	-	-	-	7.09	-	-	-	-	27	-	-	-	-	0.01	-	-	-	-	101.
**Tyre track**	-	7.11	-	-	7.16	-	32	-	-	32.40	-	0.45	-	-	0.76	-	112	-	-
**Furrow**	-	-	-	-	7.31	-	-	-	-	31.19	-	-	-	-	0.31	-	-	-	-

HS = High socioeconomic, IUF = Irrigated urban farming, LS = Low socioeconomic, MS = Middle socioeconomic and PU = Peri-urban.

**Table 4 b:** Physicochemical characteristics of larval habitat type and site categories during the rainy season

	pH					Temperature (°C)					Salinity (ppt)					TDS/Turbidity (ppm)				
Habitat type	LS	MS	HS	PU	IUF	LS	MS	HS	PU	IUF	LS	MS	HS	PU	IUF	LS	MS	HS	PU	IUF
**Artificial pond**	-	-	-	-	7.5	-	-	-	-	26.4	-	-	-	-	0.2	-	-	-	-	154
**Natural pond**	-	-	-	7.6	7.7	-	-	-	30.3	27	-	-	-	2.2	0.3	-	-	-	933	465
**Drainage ditch**	7.5	7.0	7.8	7.6	7.5	28.3	27	27.1	27.5	26.3	0.9	1.6	0.6	0.6	0.3	1460	717	340	511.1	19
**Swamp**	8.0	9.2	8.3	7.7	7.7	25.6	27.7	28.4	28	26.1	0.2	0.9	0.5	0.4	0.3	4940	695	271.5	372.2	210
**Well**	7.3	9	7.2	8.7	-	29.7	26.3	27.3	26.6	-	1.5	0.6	0.6	1.2	-	219	898	887	220.2	-
**Footprint**	7.7	8.3	-	7.7	7.7	27	28.8	-	26.8	25.2	0.8	2	-	0.3	1.6	4760	0	-	317.4	259
**Tyre track**	7.6	8	8.3	7.8	7.7	26	28.7	27.3	27.8	27.1	0.4	1.4	0.6	0.5	0.9	900	637	573.6	1041	189
**Furrow**	-	-	-	-	8.5	-	-	-	-	28.7	-	-	-	-	1	-	-	-	-	50.
**Car tyre**	7.5	7.9	7.7	-	-	29.5	27.4	28.3	-	-	0.28	0.7	0.5	-	-	395	372	211	-	-
**Puddle**	-	7.3	8.3	7.5	7.4	-	29.3	29.5	29.6	29.3	-	1.6	1.1	1.0	0.9	-	569	256	0.524	564
**Hooves print**	-	-	-	7.7	-	-	-	-	28.8	-	-	-	-	1.1	-	-	-	-	468	-

HS = High socioeconomic, IUF = Irrigated urban fa socioeconomic, PU = Peri-urban, and “-” = no larval habitat found.

**Table 5:** Correlation table showing relationship between physicochemical parameters and *An. qambiae* s.l. larval density.

	An. gambiae LD	pH	Temp	EC	Salinity	TDS
An. gambiae LD	1.00000000	−0.05842815	0.09015693	0.21804142	0.22186362	0.22775660
pH	−0.05842815	1.00000000	0.02301607	−0.09329249	−0.09390100	−0.08588305
Temp	0.09015693	0.02301607	1.00000000	0.11895627	0.12155853	0.12887996
EC	0.21804142	−0.09329249	0.11895627	1.00000000	0.90719215	0.88264834
Salinity	0.22186362	−0.09390100	0.12155853	0.90719215	1.00000000	0.9082542
TDS	0.22775660	−0.08588305	0.12887996	0.88264834	0.9082542	1.00000000

**Table 6:** Level of physicochemical parameters of polluted and unpolluted larval habitats

	Nima		Chorkor		Teshie		Madina	
Physicochemical Parameters	Polluted	Unpolluted	Polluted	Unpolluted	Polluted	Unpolluted	Polluted	Unpolluted
Temperature (°C)	27.3	28.7	28.4	28.3	26.8	27.5	28	28
Pressure (mmHg)	757.7	759.0	762.2	762.3	760.9	760.5	756.2	757.7
DO (%)	17.6	26.1	36.95	51.4	46.3	100	34.2	34.1
DO (ppm)	1.4	2.0	2.9	3.96	3.6	7.8	2.7	2.7
SPC (mS/cm)	7.0	2.7	2.93	2.1	8.05	1.5	1.6	1.2
SPC (uS/cm)	7332.5	2932.0	3114.7	2237	8319.7	1807	1657.2	1219.80
Salinity (ppt)	3.9	1.4	1.5	1.1	4.4	0.9	0.8	0.6
TDS (mg/L)	4567.0	1780.2	1901.5	1366.8	5229.3	1120.7	1026.5	751
Resistivity (ohm-cm)	142.3	366.3	2750.4	475.7	124.3	580	704.3	876.3
pH	8.3	7.8	8.56	8.3	9.1	8.3	8.4	7.8
NH4-N(mg/L)	11.9	4.9	0.4	0	1.14	0	0.1	0.11
NH3-N(mg/L)	1.6	0.2	0.04	0	0.18	0	0	0
**Larval density**	**2.6**	**1.2**	**6.6**	**4.5**	**4.7**	**1.9**	**3.1**	**1.7**

**Table 7:** Distribution of larval *Anopheles* species in study sites

Categories	Study Site	*An. coluzzii*	*An. gambiae* s.s.	Hybrid	*An. stephensi*	Total
**HS**	Cantonment	4	7	0	0	**11**
	East Legon	10	38	2	0	**50**
	Tantra Hills	36	14	0	0	**50**
**MS**	Madina	18	31	1	0	**50**
	Dansoman	46	2	1	1	**50**
	Teshie	38	12	0	0	**50**
**LS**	Chorkor	49	1	0	0	**50**
	Nima	36	13	1	1	**51**
	New Fadama	42	8	0	0	**50**
**IUF**	Tuba	36	9	5	1	**51**
	Dzorwulu	11	5	0	0	**16**
	Opeibea	16	34	0	0	**50**
**PU**	E. Legon Hills	15	28	7	0	**50**
	Medea	4	46	0	0	**50**
	Oyarifa	11	37	2	0	**50**
**Total**		**372**	**285**	**19**	**3**	**679**

HS = High socioeconomic, IUF = Irrigated urban farming, LS = Low socioeconomic, MS = Middle socioeconomic and PU = Peri-urban.

## Data Availability

The datasets utilized and analyzed in this study is available and can be obtained from the corresponding author upon request.

## Abbreviations

LS: Lower-socioeconomic status
MS: Middle-socioeconomic status
HS: High-socioeconomic status
PU: Peri-urban
IUF: Irrigated urban farming
PCR: Polymerase chain reaction
LSM: Larval source management
RFLP: Restriction fragment length polymorphism
